# Molecular pharmacokinetic mechanism of JBP485 against aristolochic acid I (AAI) -induced nephrotoxicity

**DOI:** 10.3389/fphar.2025.1577942

**Published:** 2025-04-30

**Authors:** Chong Wang, Huan Jin, Changyuan Wang, Jingjing Wu, Qiang Meng, Ming Zhong, Huijun Sun, Yuheng Wei, Ge Gao, Taiichi Kaku, Xiaokui Huo, Kexin Liu

**Affiliations:** ^1^ Department of Clinical Pharmacology, College of Pharmacy, Dalian Medical University, Dalian, China; ^2^ Provincial Key Laboratory for Pharmacokinetics and Transport, Liaoning Dalian Medical University, Dalian, Liaoning, China; ^3^ Pharmaceutical Research Center, Second Affiliated Hospital, Dalian Medical University, Dalian, China; ^4^ Japan Bioproducts Industry Co. Ltd., Tomigaya, Shibuya-ku, Tokyo, Japan

**Keywords:** JBP485, aristolochic acid I, OATs, nephrotoxicity, DDI

## Abstract

**Introduction:**

In this study, we investigated the protective effect of JBP485 against aristolochic acid I (AAI)-induced nephrotoxicity and explored the pharmacokinetic mechanisms. The effects of JBP485 on AAI-induced cytotoxicity and nephrotoxicity were evaluated *in vitro* and *in vivo*, respectively.

**Methods:**

To ascertain the protective effect of JBP485 against AAI-induced nephrotoxicity, we measured levels of urea nitrogen (BUN), creatinine (CRE), and indoxol sulfate in blood and urine; determined kidney weight-to-body weight ratio; and performed hematoxylin and eosin (H&E) staining. Cell viability and Western blotting assays, along with determination of malondialdehyde (MDA), superoxide dismutase (SOD), and intracellular reactive oxygen species (ROS) contents, were carried out to explore mechanisms underlying the protective effects of JBP485 against AAI-induced nephrotoxicity.

**Results:**

JBP485 treatment attenuated AAI-induced injuries in rat kidney while decreasing the levels of indoxyl sulfate, CRE, and BUN in plasma and increasing those of indoxyl sulfate in urine compared to that in AAI alone-treated group. The co-administration of JBP485 with AAI significantly increased the concentration and AUC of AAI in plasma, while decreasing its cumulative urinary excretion and renal clearance. Moreover, JBP485 reduced the uptake of AAI in kidney slices and human organic anion transporter 1/3 (hOAT1/3)-transfected human embryonic kidney 293 (HEK293) cells, suggesting that JBP485 ameliorated AAI-induced nephrotoxicity by reducing renal exposure to AAI via OAT inhibition. Meanwhile, JBP485 modulated the abnormal expressions of Oat1, Oat3, organic cation transporter 2 (Oct2), P-glycoprotein (P-gp), multidrug resistance-associated protein 2 (Mrp2) and multidrug and toxin extrusion proteins 1 (Mate 1) in rat kidney, suggesting that JBP485 improved tubular secretion in AAI-treated rats. Moreover, JBP485 reversed the AAI-induced changes in the expression of heme oxygenase 1 (HO-1), NAD(P) H: quinone oxidoreductase-1 (NQO1), B-cell lymphoma-2 (Bcl-2) protein expressions and Bcl-2-like protein 4 (Bax) induced by AAI in rat kidney. JBP485 increased cell viability and reduced intracellular levels of ROS in NRK-52E cells treated with AAI.

**Discussion:**

These results suggested that JBP485 protected against AAI-induced renal oxidative stress. All results indicated that JBP485 protected against AAI-induced nephrotoxicity by reducing renal exposure to AAI and alleviating oxidative stress. Our findings suggested that JBP485 has potential as a renoprotective agent for the prevention of AAI-induced nephrotoxicity.

## 1 Introduction

Aristolochic acids (AAs) is a general term for a class of nitrophenanthrene carboxylic acids. It contains multiple components, and Aristolochic Acid I (AAI) is one of the main and highly bioactive components, which is widely present in the medicinal herbs of Aristolochiaceae plants. These herbs have been used globally to treat various diseases for decades (Heinrich et al., 2009). In some African countries, Aristolochiaceae plants are used in traditional medicine as a gastric stimulant and for the treatment of cancer, lung inflammation, dysentery, and snake bites (Aigbe et al., 2019; Negi et al., 2003). Nephropathy caused by AAs has now been widely recognized worldwide (Pozdzik et al., 2008), characterized by acute kidney injury, progressive kidney fibrosis, and upper tract urothelial carcinoma. The toxic components are structurally related nitrophenanthrene carboxylic acids, i.e., Aristolochic Acid I (AAI) and II (AAII). AAI is considered to be the major cause of Aristolochic acid nephropathy, characterized by severe renal fibrosis and upper urothelial cancer (Bastek et al., 2019). In the research on the toxicity and carcinogenicity of AAI, human kidney diseases associated with AA exposure have attracted significant attention. These diseases are collectively referred to as “Aristolochic acid nephropathy” (AAN). There are two main causative factors. One is the ingestion of plants containing AA as part of traditional phytotherapies, which was previously known as “Chinese herbs nephropathy”. The other is environmental contaminants in food, such as those causing Balkan endemic nephropathy (Jadot et al., 2017). In humans, AAN pathology is primarily characterized by extensive cortical tubular atrophy, tubule loss, and severe interstitial fibrosis. These lesions are particularly prominent in the outer area of the cortex, with glomeruli being relatively less affected (Jelakovi et al., 2011; Depierreux et al., 1994). Approximately 5% of patients were shown to present with nonoliguric acute kidney injury and developed acute or subacute renal failure, often due to the continuous or excessive use of Chinese medicine decoctions containing AA in a short period of time (Zhou et al., 2023). As the disease progresses, progressive renal fibrosis leads to a gradual loss of kidney function, eventually developing into end-stage renal disease and kidney failure. At this point, patients often rely on dialysis or kidney transplantation to sustain their lives. AAN not only severely impacts the quality of life of affected patients but also poses a challenge to public health services, highlighting the importance of in-depth research on the mechanisms underlying AAI-induced toxicity and the development of strategies for the prevention of AAN. AAI is also a well-established carcinogen, with a strong association with the development of urothelial carcinoma (Dong et al., 2023; Huang et al., 2018). Epidemiological studies have consistently shown a strong correlation between the consumption of herbs containing AAI and the increased incidence of urothelial carcinoma in exposed populations (Yun et al., 2013). Further research on AAN contributes to a comprehensive understanding of the harm of AAI to human health.

Renal transporters are membrane proteins involved in the transport of a wide variety of compounds through biological membranes. They can be divided into uptake transporters (e.g., organic anion transporters [OATs] and organic cation transporters [OCTs]) and efflux transporters (e.g., multidrug resistance-associated proteins [MRPs], P-glycoprotein [P-gp], and multidrug and toxin extrusion proteins [MATEs]) and play an important role in the toxicokinetics of many endo- and xenobiotics. The function of these transporters may be dysregulated by renal injury, resulting in reduced toxin excretion and the exacerbation of renal damage.

OATs are mainly expressed in the kidney and are involved in the elimination of a wide range of endogenous and exogenous substances and their metabolites from the body (Sekine et al., 2000). It has been shown that the renal uptake of AAI, the most abundant and nephrotoxic AA, is mediated by OAT1 and OAT3 (Ma et al., 2021). The renal accumulation of AAI, along with nephrotoxicity, was significantly reduced in Oat1 and Oat3 gene knockout mice compared with that in their wild-type counterparts Furthermore, the co-administration of AAI with probenecid, a typical OAT inhibitor (Shibutani et al., 2007; Xue et al., 2011; Zeng et al., 2012) or rhein, an inhibitor of OAT1 and OAT3 (Ma et al., 2014), alleviated AAN (Ma et al., 2021). Above studies suggest that the uptake of AAI *via* OAT1 and OAT3 is a key factor in AAN development.

JBP485 (cyclo-trans-4-L-hydroxyprolyl-L-serine), a dipeptide with significant antihepatitic, antioxidant and antiapoptotic properties (Liu et al., 2011; Wang et al., 2011; Wu et al., 2008; Yang et al., 2009), was first isolated from Laennec, which is a hydrolysate of the human placenta. We previously demonstrated that JBP485 attenuates drug-induced kidney injury by modulating the expression of OATs and MRP2 (Guo et al., 2013; Liu et al., 2012). Moreover, JBP485 can inhibit the OAT1-and OAT3-mediated renal uptake of imipenem (IMP), thereby alleviating its nephrotoxicity (Wang et al., 2022). This suggests that the combination of the antiapoptotic, antioxidant, and OAT-inhibitory properties of JBP485 may exert modulatory effects in AAN.

Given the above observations, in this study, we investigated whether JBP485 can moderate AAN and sought to clarify the potential mechanism involved in drug-drug interactions (DDIs) between AAI and JBP485. We found that JBP485 ameliorated AAN by reducing renal exposure to AAI, restoring renal secretory function, and alleviating oxidative stress. These findings provide a novel approach to reducing the risks associated with AAN in clinical settings.

## 2 Materials and methods

### 2.1 Materials

AAI was purchased from Nanjing DASF Biological Technology Co., Ltd. JBP485 was supplied by Japan Bioproducts Industry Co., Ltd. (Tokyo, Japan). Indoxyl sulfate was obtained from Sigma-Aldrich (St. Louis, MO, United States). Kits for the determination of malondialdehyde (MDA), superoxide dismutase (SOD), creatinine (CRE), and blood urea nitrogen (BUN) contents were purchased from Nanjing Jiancheng Bioengineering Institute (Nanjing, China). All other reagents and chemicals utilized in this study were of analytical grade and were commercially available.

### 2.2 Animals

Male Wistar rats, weighing 220–250 g, were obtained from the Experimental Animal Centre of Dalian Medical University (Dalian, China; permit number SCXK 2013–003). All animals were fed a chow diet and allowed free access to water. All animal experiments were performed according to the guidelines for the care and use of laboratory animals of the National Institutes of Health. The animals were fasted for 12 h before pharmacokinetic experiments were performed.

### 2.3 Cell culture

The normal rat kidney epithelial cell line (NRK-52E) and human embryonic kidney 293 (HEK293) cells were grown in Dulbecco’s modified Eagle’s medium (DMEM; Invitrogen, United States) supplemented with 10% (v/v) fetal bovine serum (FBS), 100 U/mL penicillin, and 100 mg/mL streptomycin. The cells were cultured at 37°C with 5% (v/v) CO_2_ and 95% relative humidity. Cell culture reagents were purchased from Gibco (Grand Island, NY, United States).

### 2.4 Toxicity study in rats

For the assessment of toxicity, AAI was suspended in olive oil and JBP485 was dissolved in saline. Rats were randomly divided into the following four groups (n = 5 per group): A control group, which was administered saline intraperitoneally (i.p.) and olive oil orally (p.o.); a JBP485-only group, administered JBP485 i. p. at the dose of 50 mg/kg and olive oil p. o.; an AAI-only group, in which the rats were administered AAI p. o. at the dose of 20 mg/kg and saline i. p.; and a JBP485 + AAI group, where the animals were given AAI p. o. at the dose of 20 mg/kg and JBP485 i. p. at the dose of 50 mg/kg. JBP485 was intraperitoneally administered 2 days before AAI treatment. On day 3 and 30 min after AAI administration, rats were given saline i. p. or JBP485 (50 mg/kg) once daily for 7 days. On day 10, the rats were euthanized and blood, urine, and kidneys were collected for subsequent assays.

### 2.5 Biochemical assay

Indoxyl sulfate levels in plasma and urine of rats were determined by liquid chromatography-tandem mass spectrometry (LC–MS/MS) (API 3200; Applied Biosystems, Foster City, CA, United States) according to our previous study (Guo et al., 2013).

The levels of BUN and CRE in rat plasma were detected following the instructions of the Nanjing Jiancheng Institute of Biotechnology (Nanjing, China). Samples and reagents were added according to the instructions of the kit and reactions were carried out under the specified conditions. Absorbance was measured at 520 nm and 546 nm, respectively, and the quantities were calculated based on the corresponding formula.

### 2.6 AAI-induced cell injury

AAI was prepared to make a series of working dilutions in serum-free DMEM. The NRK-52E cells were plated in 96-well plates at a density of 5 ×10^4^ cells/mL for 24 h at 37 ^°^C and then challenged with various concentrations of AAI (0, 1, 2, 4, 8, 16, 32, 64, 128 and 256 μM). Cell viability was assessed using the CCK-8 assay.

### 2.7 Cell viability assay

NRK-52E cells were seeded in 96-well plates at a density of 5 × 10^4^ cells/mL and incubated for 24 h at 37 ^°^C. Following pretreatment with various concentrations of JBP485 (7.8125, 15.625, 31.25, 62.5, 125, and 250 μM) for 2.5 h, the cells were challenged with AAI (11 μM) for 24 h. Cell viability was subsequently assayed using a CCK-8 kit.

### 2.8 Measurement of intracellular ROS level

NRK-52E cells were plated in 6-well culture plates at a density of 5 ×10^4^ cells/mL and treated with various concentrations of JBP485 (62.5, 125 and 250 μM) for 2.5 h. After challenge with AAI (11 μM) for 24 h, the cells were harvested and resuspended in 500 μL of DCFH-DA (10 μM) for the detection of ROS by flow cytometry (Becton Dickinson, United States) (Aagaard et al., 2014).

### 2.9 Western blotting

For the extraction of total protein, 100 mg of fresh kidney tissue was placed in 1 mL of RIPA buffer containing protease inhibitor, phosphatase inhibitor, and PMSF, cut into small pieces with ophthalmic scissors, homogenized, and placed at 4°C to fully lyse for 20 min, with vortexing every 5 min. Following centrifugation at 12,000 rpm for 20 min at 4°C, the protein concentration in the supernatant was determined using a BCA protein quantification kit. The protein concentrations of all the samples were then adjusted to the lowest protein concentration measured. Subsequently, an appropriate amount of 5× loading buffer was added according to the volume, and the samples were placed in boiling water for 10 min. Equal amounts of protein (40 μg) were separated by SDS–PAGE at constant current (120 mA), transferred to a PVDF membrane by wet transfer, blocked in 5% non-fat dry milk for 2 h, and incubated overnight with primary antibodies. The next day, the samples were incubated with the corresponding secondary antibodies (goat anti-rabbit IgG [H&L], 1:2000, or goat anti-mouse IgG [H&L], 1:2000) for 2 h at room temperature. The protein bands were developed using enhanced chemiluminescence (ECL, YH0009-1, Nanjing Crane Biopharmaceuticals, Nanjing, China) and Tanon-5200 system (Shanghai Tianneng Technology Company, Shanghai, China) while ImageJ 6.0 software was used for analysis.

### 2.10 *In vivo* renal clearance experiments

Rats were randomly assigned to an AAI (4 mg/kg) group or an AAI (4 mg/kg) + JBP485 (50 mg/kg) group (n = 5 per group). The drugs were diluted in normal saline and administered intravenously via the left jugular vein. Subsequently, blood samples (0.2 mL) were collected via the right jugular vein with heparinized syringes at 1, 3, 5, 10, 30, 60, 120, 240, 360, and 480 min post-treatment. Isotonic saline solution (0.2 mL) was injected after each blood sample collection. Urine was collected directly from the bladder at 2, 4, 6, and 8 h after drug administration. AAI concentrations were measured by LC–MS/MS. Pharmacokinetic parameters, renal clearance (CL_R_), and cumulative urinary excretion were calculated.

### 2.11 *In vitro* uptake in kidney slices

Rat kidneys were cut into slices with a ZQP-86 tissue slicer (Zhixin Co. Ltd., China), as previously described (Wang et al., 2014). After preincubation for 3 min at 37°C, the kidney slices were transferred to 24-well culture plates containing 1 mL of fresh oxygenated buffer with AAI (1 μM) and/or JBP485 (500 μM) and further incubated at 37 ^°^C with gentle shaking. After incubation for 5, 15, and 30 min, the slices were washed with ice-cold Hanks’ balanced salt solution (pH 7.5). The amount of AAI accumulated in the homogenized kidneys was determined by LC–MS/MS.

### 2.12 Uptake by OAT1/3-transfected HEK293 cells

Uptake experiments with hOAT1/3-HEK293 cells were performed as previously described (Zhu et al., 2020). Uptake was initiated by adding transport buffer (1 mL) with AAI (1 μM) and/or JBP485 (500 μM) after the cells were washed twice and incubated with transport buffer at 37°C. Moreover, the concentration-dependent uptake of AAI and the effects of JBP485 on AAI uptake were examined. Uptake was terminated upon the removal of the medium. The cells were washed three times with 1 mL of ice-cold Hanks’ balanced salt solution, lysed with 0.3 mL of 0.1% (v/v) Triton X-100, and then transferred to a polythene tube for quantification by LC-MS/MS. The protein level was measured using the BCA method (Solarbio, Beijing, China) with bovine serum albumin serving as the standard.

### 2.13 LC-MS/MS analysis

The concentrations of the test compounds in plasma, urine, and cell lysates were determined using an Agilent HP1200 liquid chromatography system (Agilent Technology Inc., SC, CA, United States) and an API 3200 triple-quadrupole mass spectrometer (Applied Biosystems, SC, CA, United States). After protein precipitation using acetonitrile, the analyte was separated on a Hypersil BDS C18 column. The mobile phase consisted of acetonitrile and water containing 0.1% (*v*/*v*) formic acid (80:20, *v*/*v*). The flow rate was 0.4 mL/min. The mass spectrometer was equipped with an electrospray ionization (ESI) source. Data were acquired in positive ion mode and dynamic multiple reaction monitoring (MRM) mode. The ESI source conditions were as follows: ion spray voltage, 5.5 kV; ion source temperature, 600°C; Gas source 1 (GS1, N2) pressure, 50 psi; Gas source 2 (GS2, N2) pressure, 60 psi; curtain gas (CUR) pressure, 12 psi; and collision gas (CAD) pressure, 10 psi. EP and CXP were set at −10 eV for all analytes. The scan range was *m*/*z* 359.9→298.1 for AAI and *m*/*z* 130.0→71.2 for IS (Internal Standard, metformin). A lower limit of quantification of 1 ng/mL was achieved for AAI, with precision and accuracy being within acceptable limits. The method showed linearity over the concentration range of 1–1,000 ng/mL for AAI. Intra- and inter-day precision was lower than 4.5% with reference to relative standard deviation, while accuracy was within ±6.5% in terms of relative error for analytes. Analyst software (version 1.4.1) was used for equipment control and data acquisition and analysis.

### 2.14 Data analysis

Statistical analysis was performed using SPSS 13.0 software. All data are presented as means ± standard deviation (SD). One-way analysis of variance (ANOVA) was used to assess the significance of differences among the various groups. In all cases, P < 0.05 was considered to be statistically significant.

## 3 Results

### 3.1 Protective effect of JBP485 against AAN in rats

**FIGURE 1:**
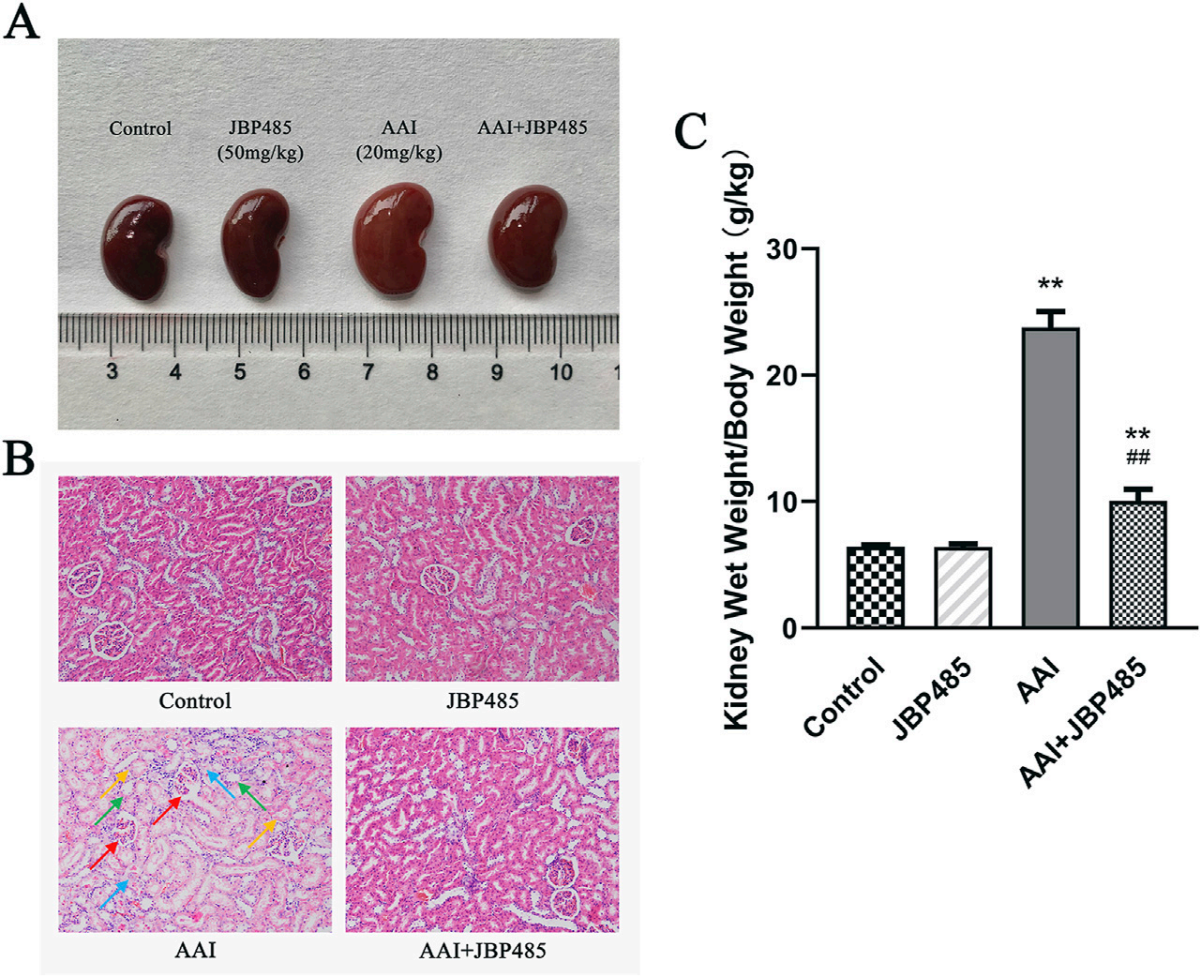
JBP485 improved the morphology and pathology condition of the kidneys in rats. The changes in morphology **(A)**, histopathology **(B)** and the kidney weight-to-body weight ratio **(C)** after co-administration of JBP485. Glomerular shrinkage (red arrows), tubule dilatation (green arrows), renal tubular epithelial cell deformation and vacuolation (blue arrows) and necrosis and shedding of cell (yellow arrows). Data are presented as mean ± S.D. ^**^p < 0.01 compared with control group; ^##^p < 0.01 compared with AAI alone-treated group (n = 5).

**FIGURE 2:**
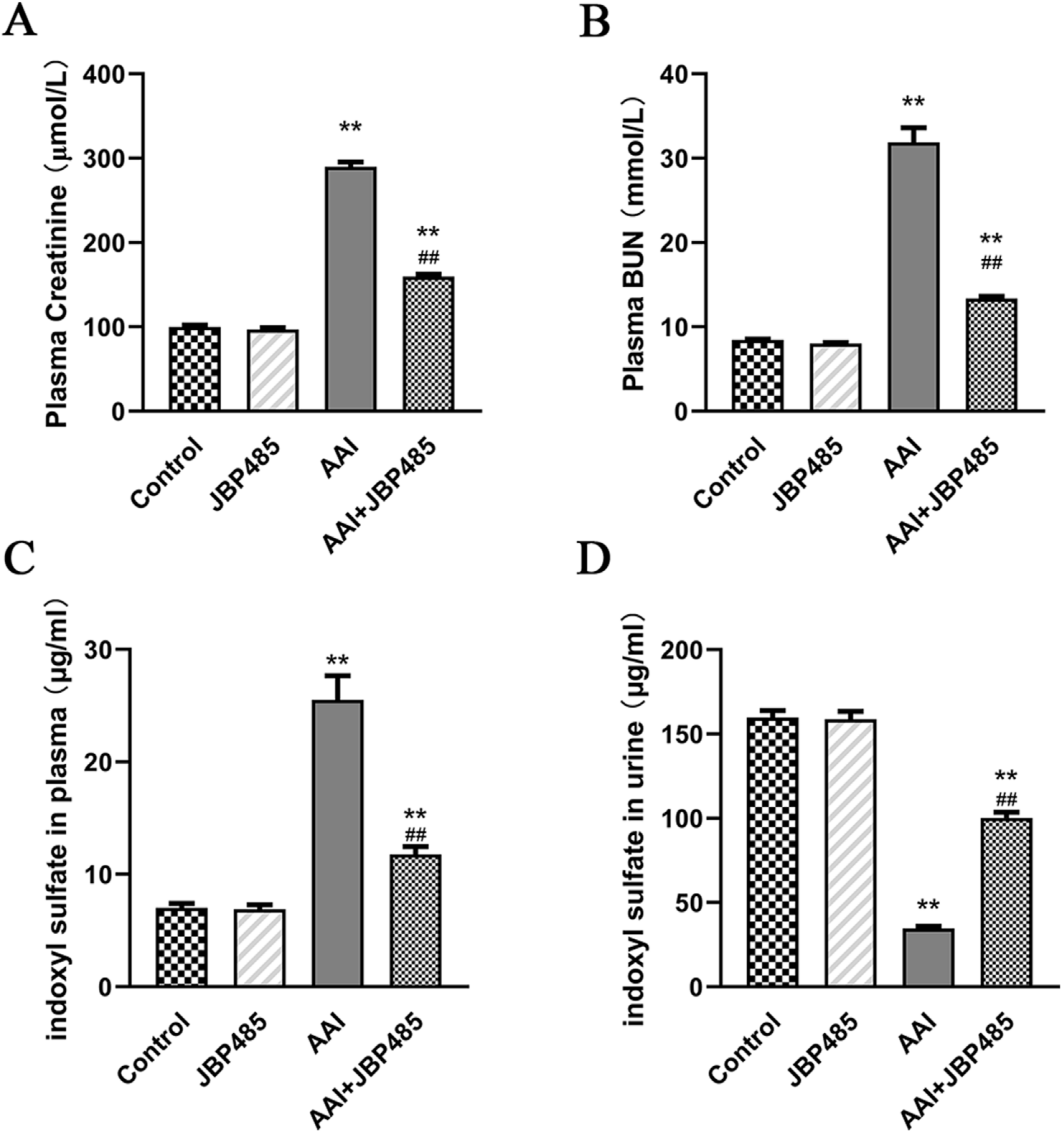
Protective effect of JBP485 on AAI nephrotoxicity *in vivo*. Blood and urine samples were collected for the determination of CRE **(A)**, BUN **(B)**, plasma **(C)** and urine indoxyl sulfate levels **(D)**. Data are presented as mean ± S.D. ^**^p < 0.01 compared with control group; ^##^p < 0.01 compared with AAI alone-treated group (n = 5).

AAI was administered to rats with or without JBP485 to determine whether JBP485 protects against AAN in rats. Our assessment of nephrotoxicity was based on histopathological and biochemical indicators. After the administration of AAI, body weight was significantly decreased compared with that observed with the control treatment. The kidneys of rats in the AAI group were markedly swollen and were lighter in color (Figure 1A). Treatment with JBP485 alone did not influence body weight or renal morphology. The co-administration with JBP485 significantly prevented AAI-induced weight loss and markedly improved renal morphology (Figure 1). Renal morphology was assessed using hematoxylin and eosin (H&E) staining. Regarding kidney histology, no significant difference was found between the JBP485 alone and control groups (Figure 1B). Conversely, the kidneys of rats in the AAI group showed damage, exhibiting typical histopathological findings that included degeneration of interlobular arteries, atrophy of tubular cells and tubular cell death by necrosis and apoptosis (Figure 1B). However, JBP485 co-administration significantly mitigated these AAI-induced effects in the rat kidney (Figure 1B). Compared with the control treatment, AAI administration resulted in an increase in kidney weight and the kidney weight-to-body weight ratio, alongside a decrease in body weight. The ratio was reduced with the co-administration of JBP485 (Figure 1C). Moreover, to evaluate renal function, we next measured the levels of CRE, BUN, and indoxyl sulfate in both plasma and urine (Figure 2). Compared with the control group, the plasma levels of CRE (Figure 2A), BUN (Figure 2B), and indoxyl sulfate (Figure 2C) were significantly higher in the AAI group, whereas the urine levels of indoxyl sulfate were lower (Figure 2D). Nevertheless, the co-administration of JBP485 resulted in the opposite trend (Figure 2C).

### 3.2 Protective effect of JBP485 on AAI-induced cytotoxicity in NRK-52E cells

**FIGURE 3:**
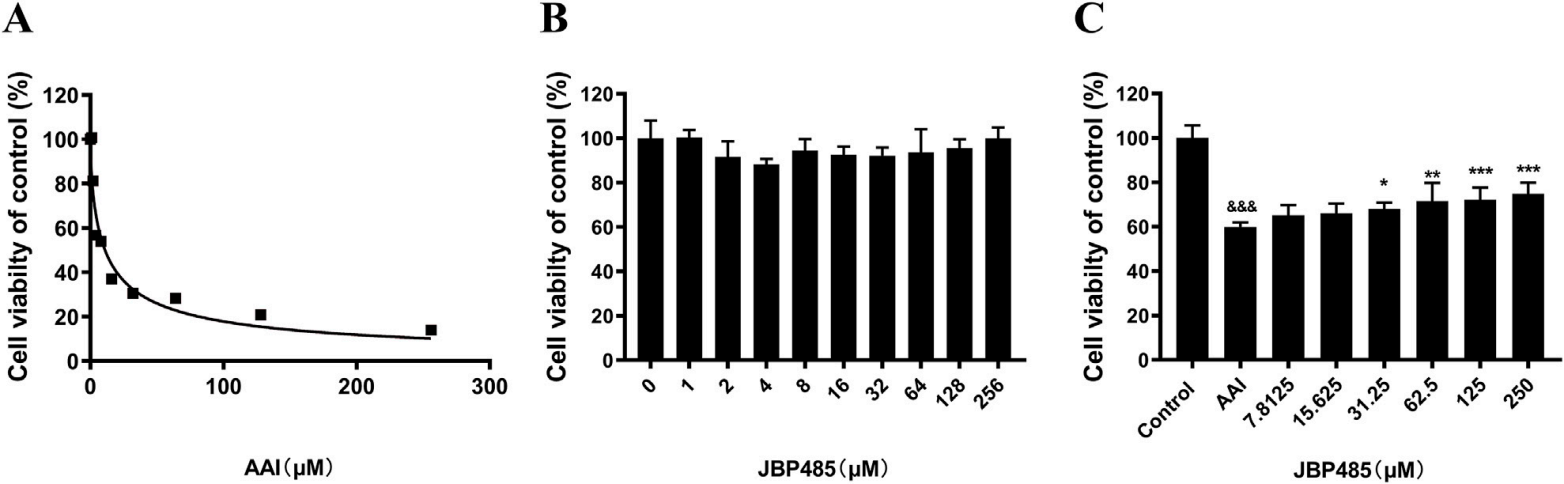
JBP485 reduced AAI cytotoxicity on NRK-52E cells. **(A)** AAI toxicity in NRK-52E cells. **(B)** JBP485 toxicity in NRK-52E cells. **(C)** Protective effect of JBP485 on AAI cytotoxicity on NRK-52E cells. Data are presented as mean ± S.D. ^&&&^p < 0.001 compared with control group; *p < 0.05, **p < 0.01 and ***p < 0.001 compared with AAI alone-treated group (n = 3).

To minimize the influence of potential confounding factors associated with *in vivo* experiments and further determine the protective effects of JBP485 on AAI-induced cytotoxicity and the related underlying mechanisms, CCK-8 assays were conducted in NRK-52E cells. The results showed that at concentrations as low as 2 μM, AAI significantly inhibited the proliferation of NRK-52E cells, while NRK-52E cell survival rates decreased in a concentration-dependent manner (Figure 3A). Furthermore, the IC_50_ value of AAI for NRK-52E cells was found to be 11 μM. Meanwhile, no JBP485-related toxicity against NRK-52E cells was detected at concentrations up to 256 μM (Figure 3B). Additionally, as shown in Figure 3C, JBP485 co-treatment dose-dependently increased the survival rates of NRK-52E cells, suggesting that it protected against AAI-induced cytotoxicity.

### 3.3 Antioxidant effects of JBP485 on AAN

**FIGURE 4:**
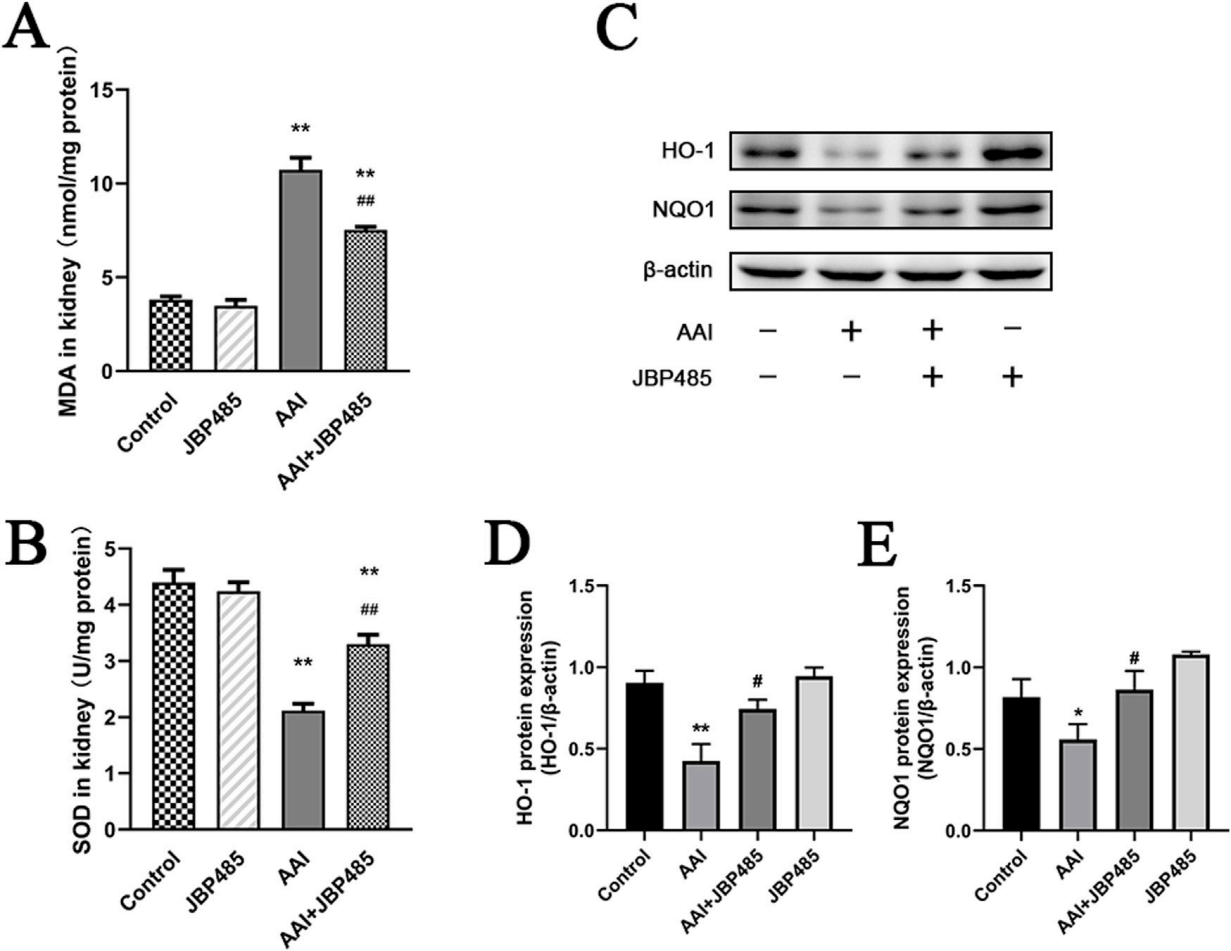
Reduction of oxidative stress in kidney tissue by JBP485. Changes in renal MDA levels **(A)**, renal SOD levels **(B)**, HO-1 and NQO1 protein expression **(C)** and statistical analysis of them Western blot images **(D, E)**. Data are presented as mean ± S.D. ^*^p < 0.05, ^**^p < 0.01 compared with control group; ^#^p < 0.05, ^##^p < 0.01 compared with AAI alone-treated group (n = 5).

To determine how JBP485 protects against AAN, we measured the levels of MDA and SOD in kidney tissues, as well as the expression of the antioxidative stress-related proteins heme oxygenase 1 (HO-1) and NAD(P) H: quinone oxidoreductase-1 (NQO1). Compared with the control group, MDA levels in the kidney were markedly increased in the AAI group (Figure 4A), while the protein levels of SOD, HO-1, and NQO1 were significantly decreased (Figures 4B, D, E). However, these effects of AAI were reversed by JBP485 treatment (Figures 4A–C), suggesting potent antioxidant effect of JBP485 in rats.

**FIGURE 5:**
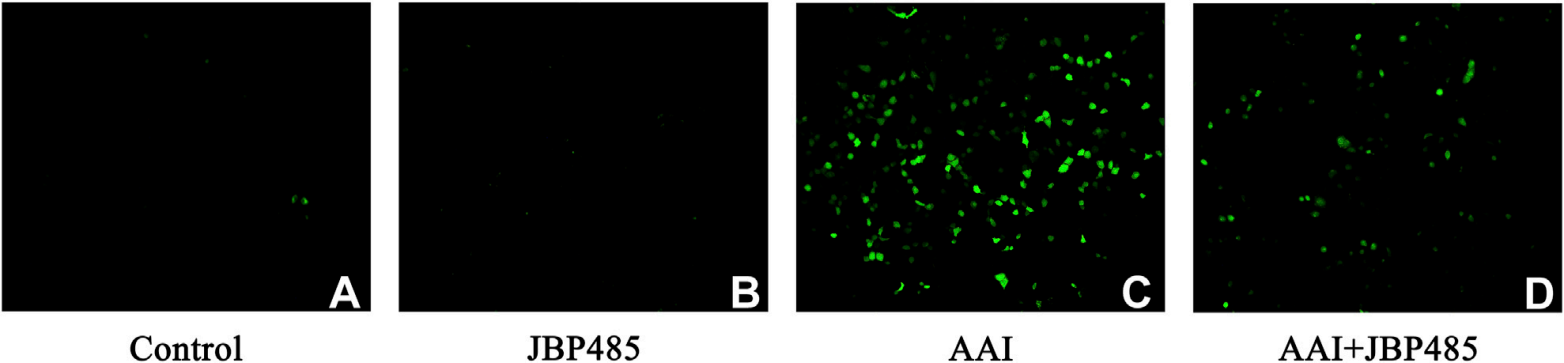
JBP485 reduced oxidative stress induced by AAI in NRK-52E cells. Control group **(A)**, JBP485 alone group **(B)**, AAI alone group **(C)**, AAI+JBP485 group **(D)** (n = 3).

We further investigated the levels of ROS in NRK-52E cells treated by AAI with or without JBP485. Compared with the control treatment, incubation with JBP485 alone did not alter ROS production in NRK-52E cells (Figure 5B), whereas a significant increase in ROS generation was recorded after treatment with AAI alone (Figure 5C). The co-administration of JBP485 significantly attenuated the rise in ROS production induced by AAI (Figure 5D). These results showed that JBP485 can effectively alleviate the damage caused by AAI-induced oxidative stress in rats and NRK-52E cells, thus protecting against AAN.

### 3.4 Anti-apoptotic effect of JBP485 in AAI-induced kidney damage

**FIGURE 6:**
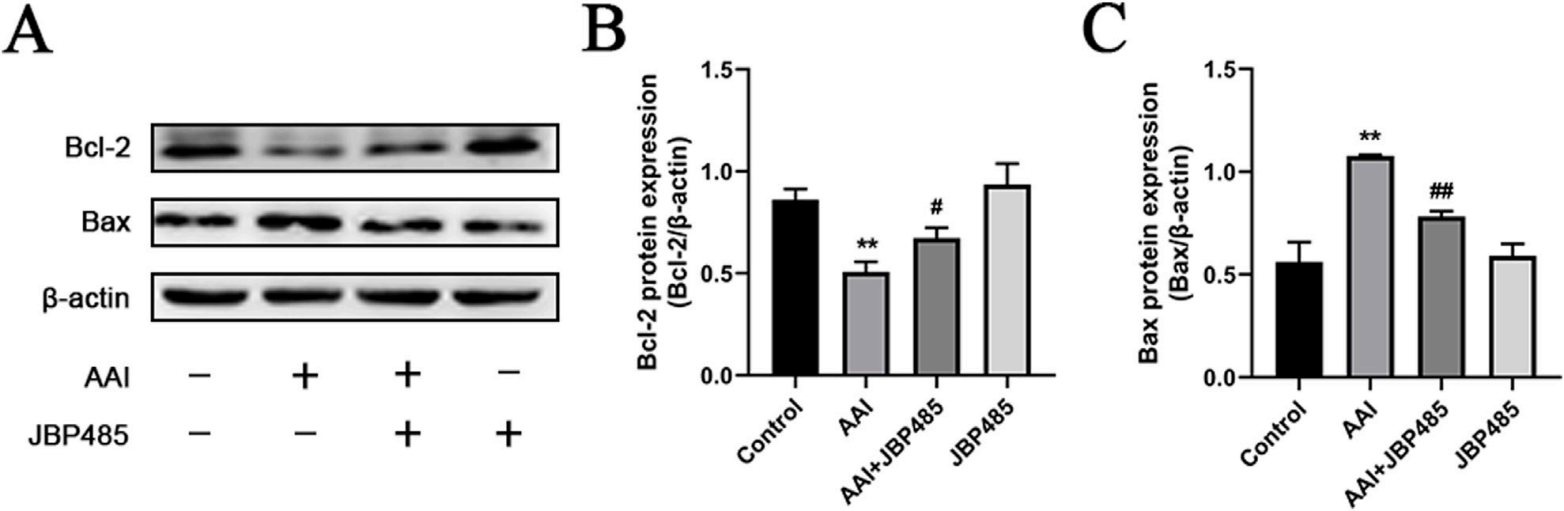
Inhibition of renal apoptosis by JBP485. Bcl-2 and Bax **(A)** protein expression and statistical analysis of them Western blot images **(B, C)**. Data are presented as mean ± S.D. ^**^p < 0.01 compared with control group; ^#^p < 0.05, ^##^p < 0.01 compared with AAI alone-treated group (n = 5).

To further evaluate the effect of JBP485 on AAI-induced apoptosis in the rat kidney, the protein expression of B-cell lymphoma-2 (Bcl-2) and Bcl-2-like protein 4 (Bax) in renal tissue was detected by Western blot. Compared with the control group, the expression levels of Bax were increased while those of Bcl-2 were decreased in the kidneys of rats in the AAI group; however, these effects were notably reversed with the co-administration of JBP485 (Figure 6). Furthermore, no change in the expression levels of the two proteins was detected in the JBP485-only treatment group. These results suggested that JBP485 inhibits AAI-induced apoptosis, thereby alleviating AAN.

### 3.5 Effect of JBP485 on renal excretion and plasma concentrations of AAI in rats

**FIGURE 7:**
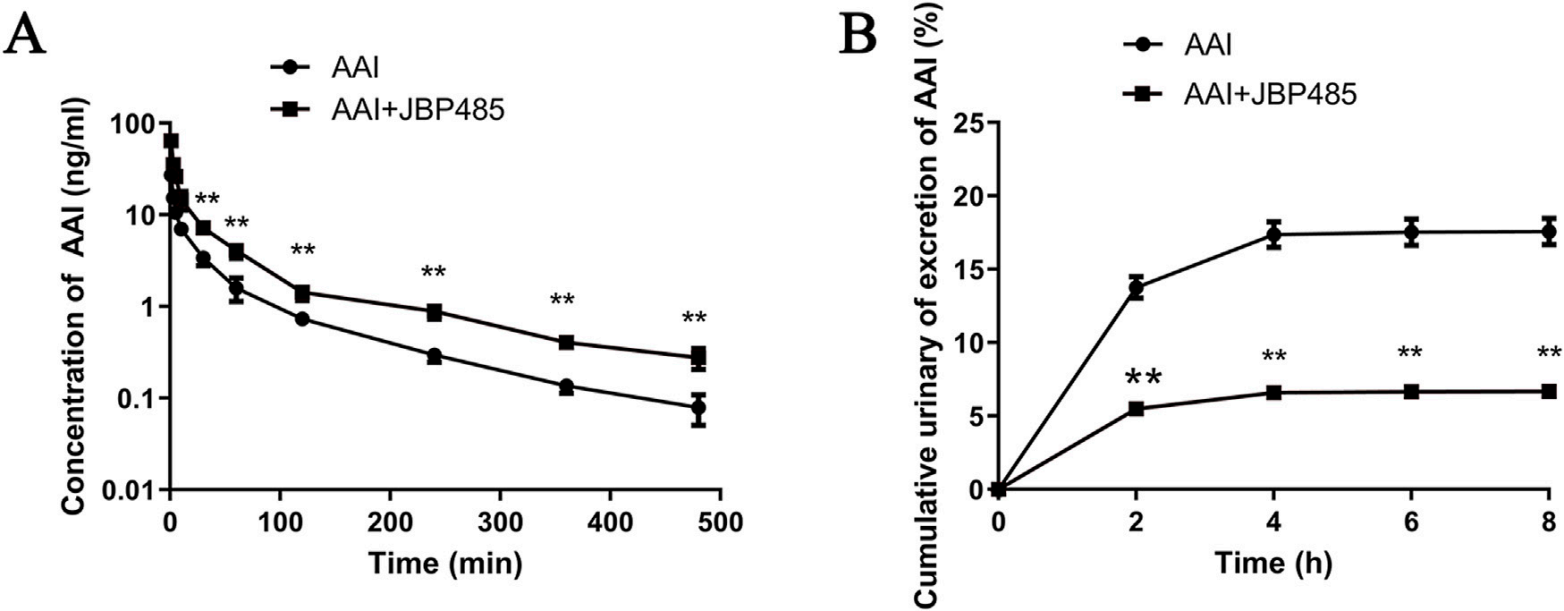
Effect of JBP485 on the pharmacokinetics of AAI in rats. Mean plasma concentration-time curves **(A)**, cumulative urine excretion curves **(B)** after intravenous administration of AAI and JBP485 in rats. Data are expressed as the mean ± SD. ^∗∗^p < 0.01 compared with control (n = 5).

The plasma concentration and the cumulative urinary excretion of AAI, with or without JBP485 co-administration, were determined to uncover the potential pharmacokinetic DDI responsible for the protective effect of JBP485 against AAN. AAI plasma concentrations, the area under the plasma concentration-time curve (AUC) and half-life (t_1/2β_) of AAI were significantly increased and plasma clearance (CL_p_) decreased in rats following the concurrent administration of JBP485 (Figure 7A; Table 1). Compared to AAI administration alone, co-treatment with JBP485 led to a reduction in the cumulative urinary excretion over 8 h and the renal clearance (CL_R_) of AAI (Figure 7B; Table 1). These findings suggested that there was a pharmacokinetic DDI between AAI and JBP485 and JBP485 inhibited renal uptake of AAI in rats.

**TABLE 1:** Pharmacokinetic parameters of AAI after intravenous administration of AAI with or without JBP485 in rats.

Parameter	Unit	AAI	AAI + JBP485
AUC(0-t)	μg/L·min	496.0 ± 14.5	1,111.7 ± 47.8^**^
AUC_0-∞_	μg/L·min	536.2 ± 14.3	1,231.3 ± 52.3^**^
t_1/2β_	min	96.6 ± 6.41	150.0 ± 12.9^**^
CL_p_	L/min/kg	7.5 ± 0.2	3.3 ± 0.1^**^
CL_R_	L/min/kg	1.24 ± 0.09	0.21 ± 0.03^**^
Vd	L/kg	232.9 ± 13.0	83.2 ± 8.15^**^

Data are expressed as the mean ± SD.

^∗∗^P < 0.01 compared with AAI, group (n = 5).

### 3.6 Effect of JBP485 on the uptake of AAI in kidney slices and hOAT1-/hOAT3-transfected HEK293 cells

**FIGURE 8:**
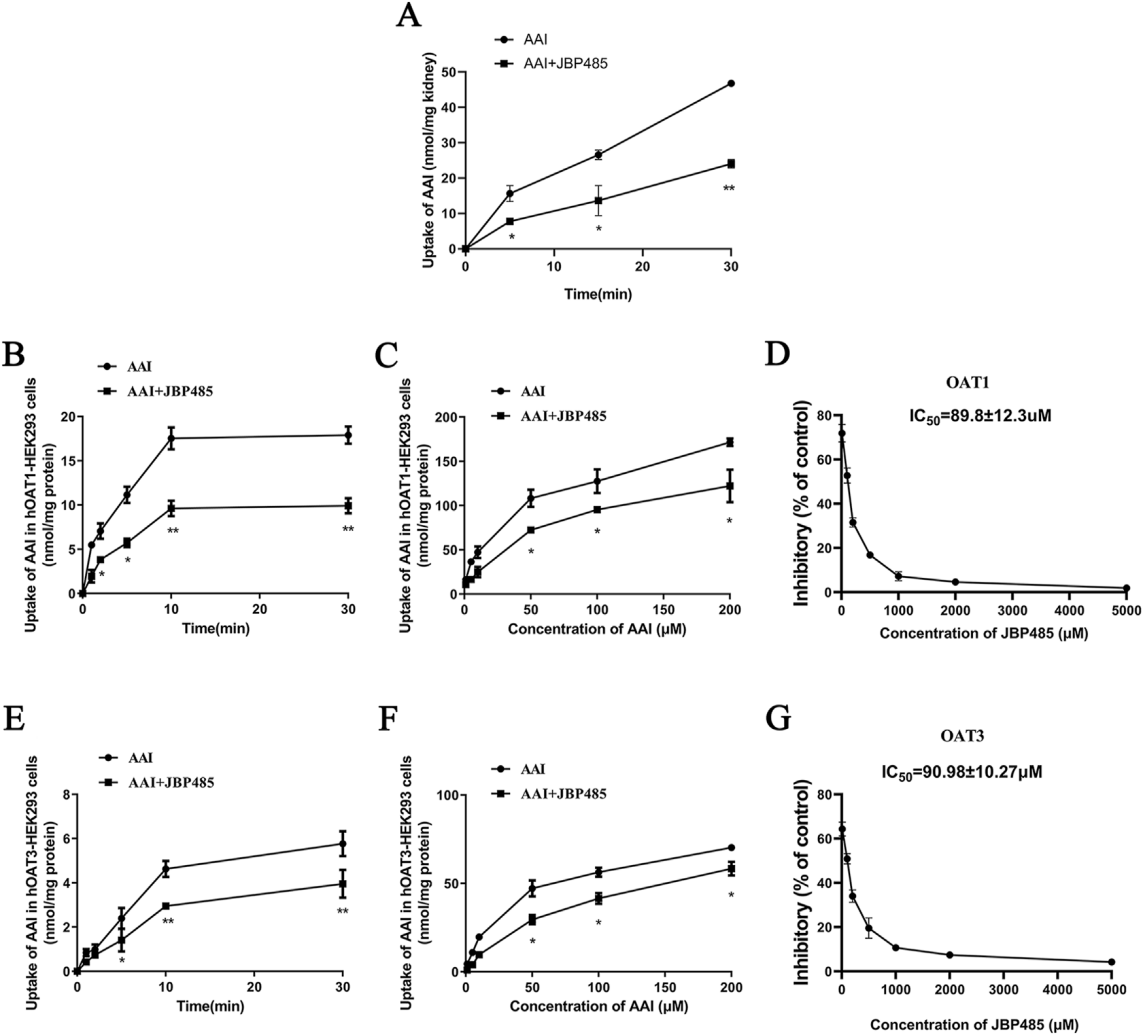
Effect of JBP485 on the uptake of AAI by rat kidney slices and hOAT1/3-HEK293 cells. Inhibition effect of JBP485 on the uptake of AAI in kidney slices **(A)**. The time-dependent inhibitory effects of JBP485 on AAI uptake in hOAT1/3-HEK293 cells **(B and E)**. The concentration-dependent inhibitory effects of JBP485 on AAI uptakein hOAT1/3-HEK293 cells **(C and F)**. The inhibitory effect of JBP485 on the uptake of AAI in hOAT1/3-HEK293 cells **(D and G)**. Data are expressed as the mean ± SD. ^∗^p < 0.05 and ^∗∗^p < 0.01 compared with control (n = 3).

Next, we sought to identify the target transporters involved in the DDI between AAI and JBP485. To exclude the impact of physiologic conditions, we used freshly prepared rat kidney slices and hOAT1-/hOAT3-transfected HEK293 cells for this experiment. We found that JBP485 inhibited AAI uptake in kidney slices (Figure 8A), suggesting that the DDIs between AAI and JBP485 occurred in the kidney. The inhibition also occurred on the AAI uptake in hOAT1-and hOAT3-transfected HEK293 cells following the addition of JBP485 (Figures 8B, E). Furthermore, JBP485 decreased the intracellular levels of AAI in hOAT1-and hOAT3-transfected HEK293 cells in a concentration-dependent manner, with IC_50_ values of 89.8 ± 12.3 μM for OAT1 and 90.98 ± 10.27 μM for OAT3 (Figures 8D, G). The uptake of AAI in hOAT1-and hOAT3- HEK293 cells was also inhibited by JBP485 at varying concentrations (Figures 8C, F). Eadie-Hofstee plot analysis showed that JBP485 significantly increased the K_m_ values of AAI uptake by hOAT1-and hOAT3-transfected HEK293 cells, whereas the V_max_ values remained unchanged (Table 2), suggesting a competitive inhibition between AAI and JBP485. These results confirmed that renal OAT1 and OAT3 were the targets of the DDI between JBP485 and AAI.

**TABLE 2:** K_m_ and V_max_ values of AAI with or without JBP485 in hOAT1-HEK293 cells and hOAT3-HEK293 cells.

Group	hOAT1-HEK293 cells		hOAT3-HEK293 cells	
	K_m_	V_max_	K_m_	V_max_
	μM	nmol/mg protein/1min	μM	nmol/mg protein/1min
AAI	15.7 ± 0.4	0.141 ± 0.04	30.6 ± 1.2	0.077 ± 0.03
AAI + JBP485	39.7 ± 0.8^∗^	0.138 ± 0.02	69.9 ± 1.4^∗^	0.075 ± 0.05

Data are expressed as the mean ± SD.

^∗^P < 0.05 compared with AAI, group (n = 3).

### 3.7 JBP485 regulated the protein expressions of Oat1, Oat3, Oct2, P-gp, Mrp2, and Mate1

**FIGURE 9:**
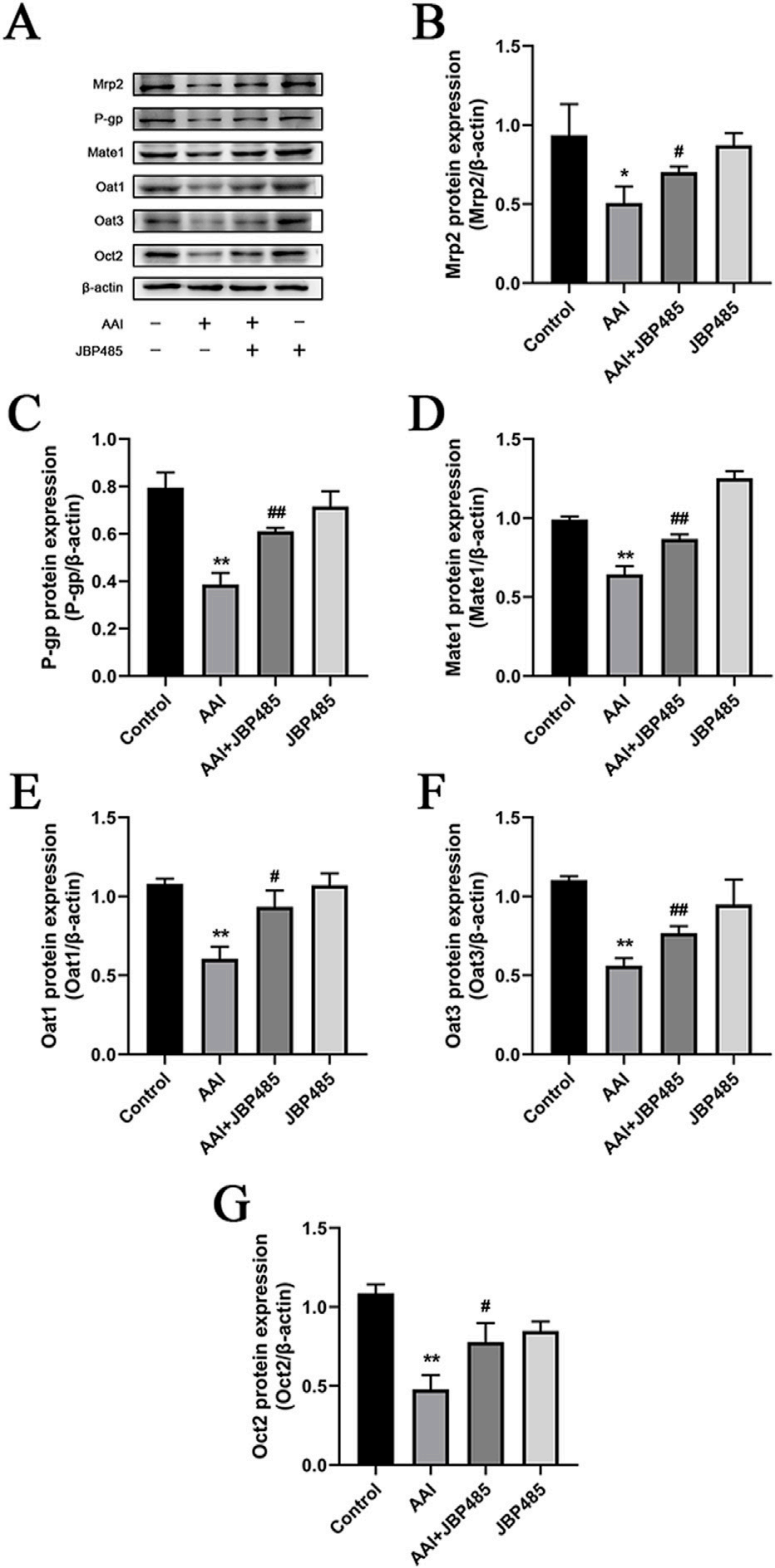
Effects of JBP485 on Mrp2, P-gp, Mate1, Oat1, Oat3 and Oct2 protein expression in the kidney. Changes on these transporters protein expression **(A)**. Statistical analysis of Western blot images of the above transporter proteins in rats **(B–G)**. Data are presented as mean ± S.D. *p < 0.05, **p < 0.01 compared with control group; ^#^p < 0.05, ^##^p < 0.01 compared with AAI alone-treated group (n = 5).

To clarify the mechanism underlying the protective effect of JBP485 on AAN, the protein expression levels of the renal uptake transporters Oat1, Oat3, and Oct2 and the renal efflux transporters P-gp, Mrp2, and Mate1 were determined in rats treated with AAI in the presence or absence of JBP485. Co-administration with JBP485, the protein expressions of above mentioned transporters were significantly increased compared with that in the AAI alone group (Figure 9). These results indicated that JBP485 reversed the AAI-induced downregulation of renal transporter expression, thereby promoting endotoxin excretion and alleviating AAN.

## 4 Discussion

AAs are carcinogenic and nephrotoxic compounds found in various natural herbs, especially those of the Aristolochiaceae family. This plant family contains more than 500 species, a significant proportion of which are used as herbal medicines in many regions of the world (Zeng et al., 2012). In the 1990s, some women consumming slimming pills containing *Aristolochia fangchi* in Brussels, Belgium, experienced impaired renal function. AAN aroused widespread concern in worldwide (Heinrich et al., 2009). Herbs containing AAs have already been banned in many countries including the United Kingdom, Canada, Australia, Germany, and the United States. In China, proprietary Chinese medicines containing AAs are still widely used in clinical practice with strict dosing schedule (including time and dosage), such as Chuanxiling capsule, Jiming wan, Xiaoer Reke oral liquid, Xiaoke Pingchuan oral liquid. Accordingly, considerable research efforts have focused on mitigating AAN. AAI, one of the most abundant and toxic aristolochic acids, is reported to cause AAN (Feng et al., 2022), and is a substrate of OAT1 and OAT3. The accumulation of AAI in the kidney caused the nephrotoxicity (Ma et al., 2021). Co-administration of AAI with probenecid or rhein, inhibitors of both OAT1 and OAT3 (Ma et al., 2014), can reduce AAI accumulation in the kidney and protect the kidneys against AAN (Sekine et al., 2000; Shibutani et al., 2007). When orally administered, AAI downregulated Oat1/3, Oct1/2 and Octn1/2 expressions (Lou et al., 2011). JBP485 was shown to inhibit the renal uptake of OAT substrates, such as bestatin (Zhu et al., 2012), acyclovir (Ye et al., 2012) and entecavir (Xu et al., 2013). In our previous study, we found that JBP485 inhibits IMP transport through hOAT1/3, diminishes hOAT1/3-dependent cytotoxicity, and ameliorates IMP-induced nephrotoxicity (Wang et al., 2022). JBP485 exhibits significant antioxidant and antiapoptotic properties (Liu et al., 2011; Wang et al., 2011; Wu et al., 2008; Yang et al., 2009). Meanwhile, JBP485 reversed the downregulation of related renal transporter proteins expression, which was caused by kidney injury, to attenuate drug-induced nephrotoxicity (Liu et al., 2012; Zeng et al., 2012). Combined, we hypothesized that JBP485 could inhibit the renal uptake of AAI, thereby reducing its renal accumulation and exerting antioxidant and anti-apoptotic effects. Additionally, JBP485 may reverse the associated changes in renal transporter expression caused by AAI, ultimately alleviating AAN through multiple pathways.

Based on previous studies (Lou et al., 2011), we established a rat model of AAN. Our data showed that JBP485 significantly improved AAI-induced renal dysfunction and pathological changes (Figures 1, 2). Furthermore, we noted that JBP485 exerted a protective effect against AAI-induced cytotoxicity and improved the survival rates of NRK-52E cells (Figure 3). These results indicated that JBP485 attenuated AAI -induced nephrotoxicity *in vivo* and AAI -induced cytotoxicity *in vitro*.

We previously demonstrated that JBP485 is a bioactive molecule with promising anti-inflammatory, antioxidant, and antiapoptotic activities (Wen et al., 2018; Yang et al., 2009). Therefore, in the present study, we measured the levels of MDA, SOD, HO-1, and NQO1 in the rat kidney. MDA is a marker of lipid peroxidation and oxidative stress. The reduction in MDA levels by JBP485 suggests that it mitigates oxidative damage in the kidneys, which is a key contributor to AAI-induced nephropathy (Figure 4A). SOD is an antioxidant enzyme that neutralizes superoxide radicals, thus protecting cells against oxidative stress-induced damage. In this study, we observed that SOD levels were decreased with JBP485 co-treatment, indicating that JBP485 enhances the kidney’s antioxidant defense mechanisms, counteracting the oxidative stress induced by AAI (Figure 4B). Following renal injury, HO-1 plays an important role in maintaining redox homeostasis. The increase in HO-1 expression prevents further deterioration of kidney injury (Wang et al., 2014). Here, JBP485 upregulated the expression of HO-1, suggesting that JBP485 activates pathways that alleviate oxidative stress and inflammation (Figures 4C, D). Meanwhile, NQO1 is a quinone-detoxifying enzyme that helps mitigate oxidative stress, thereby exerting cytoprotective effects. NQO1 expression was increased after JBP485 treatment, further supporting the role of JBP485 in enhancing cellular defenses against AAI-induced damage (Figures 4C, E). It has been known that oxidative stress significantly increased intracellular ROS levels and caused cell structure damage (D'Autréaux and Toledano, 2007). In our study, we specifically investigated the effect of AAI on ROS levels in the presence or absence of JBP485. The results showed that AAI treatment markedly enhanced ROS production in kidney cells. However, as shown in Figure 5, treatment with JBP485 effectively reduced the AAI - induced elevation of ROS levels. This reduction of ROS levels mediated by JBP485 is consistent with its effects on other antioxidant-related factors such as MDA, SOD, HO-1, and NQO1. Lowering ROS levels might be one of the key mechanisms through which JBP485 alleviates oxidative damage in the kidneys. The resulting upregulation of SOD, HO-1, and NQO1 expression, alongside the reduction in MDA contents, ultimately enhances the overall cellular antioxidant defense system and protects cells from AAI-induced damage. This finding not only provides additional evidence for the protective role of JBP485 against AAN but also deepens our understanding of its underlying mechanism of action in counteracting AAI - induced nephropathy.

The apoptosis of renal tubular epithelial cells represents a crucial pathological mechanism underlying acute kidney injury (AKI). This process is intricately regulated by a diverse array of factors, particularly members of the Bcl-2 family. The Bcl-2 protein family encompasses both inhibitors of apoptosis, such as Bcl-2, and promoters of apoptosis, such as Bax (Li et al., 2018). Multiple studies have demonstrated the significant role played by these proteins in cell fate determination. While elevated Bcl-2 expression improves cell survival, Bax overexpression promotes cell apoptosis and can alter the response of cells to chemotherapy, radiotherapy, or other targeted therapies (Dolka et al., 2011). In the present study, we explored how JBP485 affects the expression of apoptosis-related factors in renal tubular epithelial cells. Notably, we observed that JBP485 significantly upregulated the expression of Bcl-2 while simultaneously reducing that of Bax (Figure 6). JBP485 has been reported to alleviate both liver and kidney injury by precisely regulating the expression of Bcl-2 and Bax (Wen et al., 2018). Building on these findings, the results of the current study strongly suggest that JBP485 has the potential to relieve AAN partly by inhibiting apoptosis.

AKI results from damage to the renal tubules, which reabsorb and secrete a wide variety of endogenous and xenobiotic compounds. In many common renal insults, such as ischemia or toxic injury, tubular epithelial cells, especially those in the highly metabolically active proximal tubular segment, are predominantly affected (Isaka et al., 2011). Various anionic drugs and substances are taken up by tubular cells via multi-specific OATs, present in the basolateral membrane. It has been confirmed that AAI is a substrate of OAT1 and OAT3 (Zeng et al., 2012). Additionally, several studies have reported that OAT1/3 inhibitors reduced renal exposure to AAI, thereby protecting the kidneys from AAI-related injury (Liu et al., 2011; Wang et al., 2011; Xue et al., 2011). Accordingly, we further investigated the effect of JBP485 on AAI renal excretion. The results showed that compared with rats treated with AAI only, those co-administered JBP485 and AAI exhibited significantly higher AAI plasma concentrations, lower cumulative urinary excretion of AAI, and reduced CL_R_ and CL_p_ (Figure 7; Table 1). These findings indicated that JBP485 inhibits the renal elimination of AAI, and further implied that DDI between AAI and JBP485 might be mediated by renal OATs. To test this hypothesis, we examined the effects of JBP485 on AAI uptake in kidney slices and hOAT1/3-transfected HEK293 cells and found that, in both cases, JBP485 significantly inhibited the uptake of AAI (Figure 8). We further observed that the *K*_m_ value for AAI uptake by hOAT1/3-transfected HEK293 cells was markedly increased in the presence of JBP485, whereas the *V*_max_ value remained unchanged (Table 2). These results suggested that JBP485 competitively inhibits AAI uptake. Collectively, these findings demonstrated that the DDI between AAI and JBP485 in the kidney is mediated by hOAT1 and hOAT3. Importantly, the co-administration of AAI and JBP485 reduced renal exposure to AAI, thereby decreasing the risk of AAI-induced toxicity.

Transporters such as OATs, OCTs, MRPs, P-gp and MATEs are involved in the transport of endogenous substances, toxicants and drugs from blood to urine (Anzai et al., 2006). Moreover, following kidney injury, the transport functions of these renal transporters may be impacted. As mentioned above, OATs and MRPs are involved in the excretion of indoxyl sulfate, OCT2 is involved in the excretion of plasma creatinine, MRP2 mediates the excretion of MDA, and P-gp is involved in the excretion of endogenous toxins (Deguchi et al., 2002; Im et al., 2017; Inui et al., 2000; Lim et al., 2021; Sun et al., 2006). Here, we investigated the changes in the expression of these transporter proteins after AAI treatment in the presence or absence of JBP485 (Figure 9). The results indicated that while exposure to AAI altered in the expression of Oat1, Oat3, Oct2, P-gp, Mrp2, and Mate1, these changes were reversed with by JBP485. The restoration of tubular secretion mediated by JBP485 promoted endotoxin excretion, and protected AAN (Figure 9).

Although the safety issue of AAIs has received widespread attention, in clinical practice, AAN cases have not been effectively controlled, due to widely use of Chinese herbs containing AAs. Effective measures for the prevention and control of AAN should be developed to provide a basis for the safe use of AAs. Above all, the aim of our study was to investigate the mechanism underlying the protective role of JBP485 in AAN and provide new ideas for its safe use in the clinic.

In conclusion, we found that JBP485 alleviates AAN via two main mechanisms. First, JBP485 inhibits the OAT1/3-mediated uptake of AAI and reduces AAI accumulation in the kidney. Secondly, JBP485 exerts antioxidant and anti-apoptotic effects and reverses the AAI-induced downregulation of the expression of the uptake transporter proteins Oat1, Oat3, Oct2 and the efflux transporter proteins Mrp2, P-gp, and Mate1, thereby relieving AAN.

## Data Availability

The raw data supporting the conclusions of this article will be made available by the authors, without undue reservation.

## Abbreviations

AAs, aristolochic acids; AAI, Aristolochic acid I; AAN, aristolochic acid nephropathy; DDI, drug-drug interaction; BUN, urea nitrogen; CRE, creatinine; AUC, area under the plasma concentration–time curve; OATs, Organic anion transporters; OCTs, Organic cation transporters; MRPs, multidrug resistance-associated proteins; P-gp, P-glycoprotein; MATEs, multidrug and toxin extrusion proteins; HO-1, heme oxygenase 1; NQO1, NAD(P)H: quinone oxidoreductase-1; Bcl-2, B-cell lymphoma-2; Bax, Bcl-2-like protein 4; MDA, malondialdehyde; SOD, superoxide dismutase; NRK-52E, normal rat kidney epithelial cell line; HEK293, human embryonic kidney 293; DMEM, Dulbecco’s modified Eagle’s medium; LC-MS/MS, liquid chromatography coupled with tandem mass spectrometry.
